# Changes in serum pigment epithelium-derived factor levels after kidney transplantation in patients with end-stage renal disease

**DOI:** 10.1080/0886022X.2022.2106243

**Published:** 2022-10-10

**Authors:** Réka Szentimrei, Hajnalka Lőrincz, Anita Szentpéteri, Viktória E. Varga, Mariann Harangi, Ildikó Seres, Réka P. Szabó, Balázs Nemes, György Paragh

**Affiliations:** a Division of Metabolic Disorders, Department of Internal Medicine, Faculty of Medicine, University of Debrecen, Debrecen, Hungary; b Doctoral School of Health Sciences, Faculty of Public Health, University of Debrecen, Debrecen, Hungary; c Department of Nephrology, Faculty of Medicine, University of Debrecen, Debrecen, Hungary; d Department of Organ Transplantation, Faculty of Medicine, Institute of Surgery, University of Debrecen, Debrecen, Hungary

**Keywords:** Lipids, lipoproteins, LDL, kidney transplantation, intercellular signaling peptides and proteins

## Abstract

**Background:**

Pigment epithelium-derived factor (PEDF) is a serin protease inhibitor and a potent inhibitor of angiogenesis. Its serum level has significant associations with metabolic parameters. However, little is known about the association between PEDF levels and lipid parameters in renal transplanted (TX) patients. Therefore, our aim was to investigate the relationship between PEDF level and lipid parameters in TX patients.

**Methods:**

Seventy TX patients (47 males, 23 females, mean age 51.7 ± 12.4 years) and 34 healthy controls were enrolled. We examined the serum creatinine, C-reactive protein, fasting glucose and lipid parameters right before, then 1 and 6 months after TX. High-density lipoprotein (HDL)-associated paraoxonase-1 (PON1) activities were measured spectrophotometrically. Lipoprotein subfractions were determined by Lipoprint. PEDF and oxidized low-density liporotein (oxLDL) levels were measured by ELISA.

**Results:**

Before transplantation, patients had had a significantly higher PEDF level compared to control subjects (*p* < 0.001). One month after transplantation, their PEDF level decreased significantly reaching the healthy controls’ level, and this lower level was maintained during the 6 months follow-up period as well. The initial oxLDL level was significantly higher, while PON1 activities were significantly lower in the patient group compared to the control group. We found a significant positive correlation between PEDF and total cholesterol, low-density lipoprotein (LDL)-cholesterol, triglyceride, oxLDL and small HDL subfraction; while negative correlations were found between PEDF and mean LDL size and large HDL subfraction during the entire follow-up period.

**Conclusion:**

PEDF may play an important role in the increased oxidative stress and enhanced atherogenesis in renal transplant patients.

## Introduction

Kidney transplantation provides the highest survival benefit of all possible renal replacement therapies. Previous studies have reported that patient survival is better with renal transplantation than with maintenance hemodialysis (HD) after an increased risk of death in the early period after transplantation, including patient groups who are otherwise at increased cardiovascular (CV) risk such as diabetics and obese patients [1–3]. In fact, transplant recipients have a CV risk up to 10 times higher than the general population. Cardiovascular disease (CVD) persists as the leading cause of premature death in most kidney transplant registries. Enhanced atherogenesis after transplantation is associated mostly with conventional risk factors such as hypertension, diabetes, smoking and dyslipidemia [4]. Dyslipidemia is especially common after kidney transplantation, partly due to the effect of corticosteroids, cyclosporine, tacrolimus and mammalian target of rapamycin inhibitors [5]. The significant role of oxidation in pathogenesis of atherosclerosis is well established. These findings show that oxidative stress can induce the oxidization of lipoproteins, including low-density lipoprotein (LDL-C) and this is the first event in atherosclerosis [6]. Moreover, oxidative stress is a well-known mediator of adverse outcomes throughout the course of transplantation [7].

The long-term survival of kidney allograft has improved over the past 2 decades, which also determines the patient’ s life expectancy [8]. The presence of donor-specific antibodies (DSAs) against HLA before kidney transplantation has been variably associated with decreased long-term graft survival [9]. Despite advances in diagnosis and treatment in the past decade, anti-HLA antibody mediated rejection (ABMR) still remains the main reason for the failure of kidney transplants and the return of kidney transplant recipients to dialysis [10].

Besides the traditional cardiovascular risk factors, recent evidence has demonstrated the involvement of various antiangiogenic factors in the pathogenesis of renal dysfunction and cardiovascular complications in renal diseases [11]. Pigment epithelium-derived factor (PEDF) is a multifunctional, pleiotropic glycoprotein and a potent inhibitor of angiogenesis, which is produced by a wide range of human tissues, including the liver, the adipocytes, the retina, the kidneys, as well as the vascular tissue. It is abundant in the plasma and the serum acting *via* multiple high-affinity ligands and cell receptors, however, these mechanisms are not fully clarified yet [12]. Recently, PEDF has been recognized as a counter protein against risk factors of cardiovascular disease [13, 14] due to its antioxidant, anti-inflammatory, anti-fibrotic, and insulin-sensitizing effects [14–17].

An increasing amount of data has been published recently on the circulating levels of PEDF in kidney disease, however, these findings seem to be rather contradictory. PEDF levels in patients with end-stage renal disease treated with HD are markedly higher than that of healthy controls [18]. Furthermore, higher serum PEDF levels were significantly associated with the development of renal dysfunction, assessed as macroalbuminuria or as stages 3 or 4 chronic kidney disease (CKD) [19]. However, lower levels of predialytic PEDF have been found to be associated with an increased risk of mortality in HD patients, indicating that PEDF expression may be a response to the inflammatory and oxidative processes associated with CKD [20]. In smaller patient populations, a significant enrichment of PEDF in high-density lipoprotein (HDL) particles has been found in patients with end stage renal disease compared to healthy controls, while the abundance of PEDF was lower in the HDL of transplanted patients, especially in patients with a good graft function. Still, the exact pathophysiological role of PEDF in chronic renal disease and in kidney transplanted patients has not yet been fully accounted for.

The main HDL-associated antioxidant enzyme is human paraoxonase 1 (PON1) that is synthesized by the liver and prevents LDL form the oxidative modification. PON1 is a 43 kDa calcium-dependent promiscuity enzyme which was originally referred to as A esterase due to its arylesterase activity and later described as PON because of its ability to hydrolyze an insecticide paraoxon [21]. Recently, PON1 was determined as a hydrolytic lactonase enzyme with a potential role in the development and progression of renal diseases [22].

To date, levels of PEDF and its correlations with transplant specific factors, oxidative and lipid parameters have not been studied in kidney transplant patients. Therefore, we aimed to investigate the serum PEDF levels and the relationship between the PEDF level and these parameters in this special patient population before and 6-months after transplantation and to compare their results with those of a healthy control group. The present paper is the first long-term prospective follow-up study aimed at the complex evaluation of these cardiovascular and inflammatory markers in TX patients. We hypothesized that PEDF is significantly higher before transplantation relative to the controls’ and its circulating levels correlate with lipid parameters, especially with lipoprotein subfractions before transplantation and during the follow-up.

## Patients and methods

### Patients

Seventy kidney transplant patients (47 males and 23 females, mean age: 51.7 ± 12.4 years, body mass index (BMI): 26.3 ± 4.1 kg/m^2^) were enrolled from the Institute of Surgery, Department of Organ Transplantation, University of Debrecen. Patients had been on hemodialysis 60.68 ± 52.24 months before transplantation. According to the international guidelines, the hydration status of our HD patients was regularly checked using whole-body impedance spectroscopy (BCM), which is a validated device for the precise measurement body volume compartments (Fresenius Medical Care, Bod Hamburg, Germany (BCM) Body Composition Monitor software version: 3.3.0.1637). Patients with more than 1.5–2.0 L overhydration were excluded from our study. Except for three patients – who had donation live donor – all patients underwent cadaveric organ transplantation. We also enrolled 34 healthy volunteers from the Department of Internal Medicine, University of Debrecen in the study (14 males and 20 females, mean age: 42.5 ± 6.4 years, BMI: 24.8 ± 2.1 kg/m^2^). All participants provided a written informed consent. The study protocol was approved by the Local and Regional Ethical Committees (RKEB/IKEB:4739/2017, date of approval: 20/02/2017 and ETT/TUKEB 7324-9/2017/EÜIG). The study was carried out in accordance with the Declaration of Helsinki.

We excluded participants with a liver disease, elevated liver enzymes, endocrine diseases (thyroid and parathyroid diseases, pituitary and adrenal gland disorders, etc.), an acute infective and an autoimmune disease. Further exclusion criteria were pregnancy, lactation, current smoking, alcoholism and drug addiction. Fourteen (20%) transplanted patients were diagnosed with Type 2 diabetes mellitus. The main immunosuppression regime consisted of tacrolimus, mycophenolate mofetil, and methyl-prednisolone.

### Measurement of routine laboratory parameters

Venous blood samples were collected and centrifuged at 3500 *g* for 15 min, right before, then 1 and 6 months after renal transplantation. The routine laboratory parameters – high-sensitivity C-reactive protein (hsCRP), procalcitonin, total-cholesterol, triglyceride, HDL-cholesterol (HDL-C), LDL-cholesterol (LDL-C), glucose, creatinine, glomerular filtration rate (GFR), urea – were determined by the Central Clinical Laboratory of the University of Debrecen with commercially available standard laboratory techniques on a Cobas 6000 analyzer (Roche Ltd, Mannaheim, Germany) [23]. 0.5 mL aliquots of samples were kept frozen at −70 °C for enzyme-linked immunosorbent assay (ELISA) measurements and for HDL and LDL subfraction analysis.

### Lipoprotein subfraction analyses

HDL subfractions were analyzed by a polyacrylamide gel-electrophoresis with the Lipoprint System (Quantimetrix Corp., Redondo Beach, CA) according to the manufacturer’s instructions as previously published [23]. After electrophoresis, 10 HDL subfraction bands were determined and collected into three major classes: large (HDL1-HDL3), intermediate (HDL4-HDL7), and small (HDL8-HDL10) HDL subfractions. The cholesterol concentrations of the HDL particles were calculated with the software Lipoware (Quantimetrix Corp., Redondo Beach, CA) by multiplying the total HDL-C concentration of the samples by the relative area under the curve (AUC%) of the subfraction bands.

LDL subfractions were also detected using the Lipoprint System (Quantimetrix Corp., Redondo Beach, CA) according to the instructions of the manufacturer. AUC% for the VLDL, Midband A, B, C (comprising primarily IDL), up to seven LDL subfractions and HDL peaks were calculated by the computer software Lipoware (Quantimetrix Corp., Redondo Beach, CA). Mean LDL size was calculated with the help of the same software. The percentage of large LDL (large LDL %) was defined as the sum of the percentage of LDL1 and LDL2, whereas the percentage of small LDL (small-dense LDL %) was defined as the sum of LDL3-LDL7. Cholesterol concentrations of LDL subfractions were determined by multiplying the relative AUC of subfractions by the total cholesterol concentration of the sample. The calculated total LDL-C comprises of the sum of the cholesterol in Midbands (A, B, C) and LDL subfractions (LDL1-LDL7); and strongly correlates with the directly measured LDL-C [24].

### Measurement of human paraoxonase-1 (PON1) paraoxonase and arylesterase activities

Serum PON1 paraoxonase activity was measured on a microtiter plate by a kinetic, semi-automated method using paraoxon (O,O-diethyl-O-*p*-nitrophenyl-phosphate, Sigma Aldrich, Budapest, Hungary) as a substrate. The hydrolysis of paraoxon was followed at 405 nm at room temperature. Serum PON1 arylesterase activity was assayed with a phenylacetate substrate (Sigma Aldrich, Budapest, Hungary) and the hydrolysis of phenylacetate was monitored at 270 nm at room temperature as previously described [25].

### Oxidized LDL (oxLDL) concentration measurement

Oxidized LDL level was detected by ELISA (Mercodia AB, Uppsala, Sweden, Cat. No. 10-1143-01) with 5.5–7.3 CV% intra- and 4–6.2 CV% inter-assay precision, respectively, according to the recommendations of the manufacturers [25].

### Measurement of donor-specific antibodies (DSA)

Human leukocyte antigen (HLA) antibodies assessed further for class I and class II DSA were measured by Luminex^®^ based single bead assay.

### Determination of serum PEDF level

Human PEDF concentration was determined by a commercially available ELISA kit (BioVendor, Brno, Czech Republic, Cat. No. RD191114200R) according to the manufacturer’s instructions [26]. The intra- and inter-assay coefficients of variations were 3.6% and 5.9%, respectively.

### Statistical methods

The statistical analysis was performed by STATISTICA ver.14. (TIBCO Software Inc., Palo Alto, CA). The data were presented by a descriptive analysis (means ± standard deviation or medians [lower quartile – upper quartile]). A Kolmogorov–Smirnov test was used for testing the normality of the data distribution. The comparison of data between controls and transplanted patients was performed by an unpaired *t* test or a Mann–Whitney U test; while analyzing patient data during the follow-up was performed by repeated measures analysis of variance (ANOVA). The bivariate Pearson’s correlation was assessed to measure the strength and direction of linear relationships between pairs of continuous unrelated variables. A multiple regression analysis was performed in backward manner to determine the variables’ best predicting PEDF level. The results were considered to be significant at the level of *p* < 0.05.

## Results

The main anthropometric and laboratory parameters of the enrolled subjects are summarized in Tables 1 and 2. Transplanted patients had significantly higher serum oxLDL, triglyceride, creatinine, urea, hsCRP and glucose levels than controls; while HDL-C, LDL-C, and GFR were significantly lower. After transplantation, total cholesterol, HDL-C, LDL-C, oxLDL, triglyceride, and GFR were found to be significantly increased; but mean LDL size, creatinine, urea, and hsCRP levels were found to be significantly decreased during the follow-up. Before transplantation, patients had a significantly higher PEDF level than controls (23.88 ± 4.2 µg/ml versus 14.68 ± 3.7 µg/ml; *p* < 0.001). 1 month after transplantation, their PEDF level decreased significantly reaching the healthy controls’ level (14.9 ± 3.6 µg/ml), and this low level was maintained during the 6 months follow-up period (13.9 ± 2.8 µg/ml). We were unable to detect any gender differences between females and males either in the TX (*p* = 0.52), or in the control group (*p* = 0.28). PEDF/creatinine ratio was 80% lower in cases before TX compared to controls and although this ratio significantly increased after one month, it remained only a 50% of the controls’ value. In the DSA positive group, the PEDF level was significantly lower compared to the DSA negative patients (21.8 ± 0.5 versus 24.6 ± 1.3 µg/ml; *p* = 0.03) before transplantation.

**Table 1.** Patient characteristics.

	Transplanted patients
Number of cases (*n*)	70
Female (*n*, %)	23 (32.9)
Male (*n*, %)	47 (67.1)
Age (years)	51.7 ± 12.4
Dialysis duration (months)	60.69 ± 52.24
Donor type (cadaver, *n*, %)	67 (95.7)
Donor specific antibody (DSA) positivity (*n*, %)	19 (27.1)
Donor specific antibody (DSA) positivity before TX (*n*, %)	7 (10)
Medical history	
Diabetes mellitus (*n*, %)	14 (20)
Coronary heart disease (*n*, %)	7 (10)
Peripheral artery disease (*n*, %)	3 (4.3)
Cerebral artery disease (*n*, %)	7 (10)
Cardiomyopathy (*n*, %)	15 (21.4)
Hypertension (*n*, %)	63 (90)
Medications	
Angiotensin-converting enzyme inhibitors (*n*, %)	19 (27.1)
Angiotensin receptor blockers (*n*, %)	15 (21.4)
β-Blockers (*n*, %)	38 (54.3)
Calcium-antagonists (*n*, %)	34 (48.6)
Imidazole receptors agonists (*n*, %)	4 (5.7)
α-1 receptor blockers (*n*, %)	24 (34.3)

**Table 2.** Anthropometric and laboratory parameters of study individuals.

	Controls	Cases before TX	Cases after 1 month TX	Cases after 6 months TX	*p* values
Number of subjects (male/female)	34 (14/20)	70 (47/23)			*#*
Age (years)	42.5 ± 6.4	51.7 ± 12.4			*a*
Body mass index (kg/m^2^)	24.8 ± 2.1	26.3 ± 4.1			*ns*
Cholesterol (mmol/l)	5.05 ± 0.77	4.9 ± 1.2	5.7 ± 1.32	5.3 ± 1.3	*b, c, d*
HDL-C (mmol/l)	1.52 ± 0.43	1.15 ± 0.37	1.63 ± 0.45	1.42 ± 0.4	*a, b, c, d*
LDL-C (mmol/l)	2.92 ± 0.54	2.5 ± 0.9	3.35 ± 1.1	2.99 ± 0.96	*a, b, c, d*
Oxidized LDL (U/l)	41.3 ± 9.9	48.4 ± 13	58.6 ± 17.9	50.3 ± 11.2	*a, b, d*
Mean LDL size (nm)	27.3 (27.2–27.5)	27.2 (26.8–27.4)	27.3 (27.05–27.4)	27.1 (26.9–27.3)	*b, d*
Triglyceride (mmol/l)	1.05 (0.9–1.5)	1.7 (1.2–2.8)	1.9 (1.5–2.6)	1.7 (1.4–2.5)	*a, b, c*
Creatinine (µmol/l)	68.3 ± 14.7	685.7 ± 269.9	122 ± 42	127 ± 55.7	*a, b, c*
Urea (mmol/l)	4.7 ± 1.4	16.49 ± 7.3	8.7 ± 3	8.28 ± 3.86	*a, b, c*
GFR (ml/min/1.73 m^2^)	>90	7.97 ± 3.6	59 ± 20.45	58 ± 19.7	*a, b, c*
hsCRP (mg/l)	1.57 (0.6–3.4)	3.84 (1.5–8.5)	1.5 (0.5–3.8)	2.1 (1.05–5.6)	*a, b, c, d*
Glucose (mmol/l)	4.85 (4.5–5.1)	5.3 (4.9–6)	5.5 (5–6.6)	5.2 (4.9–5.9)	*a, b, d*
PEDF (µg/ml)	14.68 ± 3.7	23.88 ± 4.2	14.9 ± 3.6	13.9 ± 2.8	*a, b, c, d*
PEDF/creatinine ratio	0.21 (0.18–0.26)	0.04 (0.027–0.047)	0.12 (0.095–0.165)	0.12 (0.09–0.15)	*a, b, c*
PON1 paraoxonase activity (U/l)	83 (48.5–168.4)	46 (33–136)	57 (41–143.3)	55.7 (39.3–167.3)	*a, b, c*
PON1 salt stimulated activity (U/l)	169.4 (100.6–305.4)	118.2 (80.4–265)	141.9 (96.2–304)	135.4 (99.1–322)	*b, c*
PON1 arylesterase activity (U/l)	133 ± 29.7	111.8 ± 24.5	130.1 ± 28.1	132.6 ± 30.1	*a, b, c*

GFR: glomerular filtration rate; HDL: high-density lipoprotein; hsCRP: high sensitive C-reactive protein; LDL: low-density lipoprotein; PEDF: pigment epithelium-derived factor; PON1: paraoxonase-1; TX: kidney transplantation. Data are presented as mean ± standard deviation or median (upper-lower quartiles).

# indicates *p* < 0.05 in controls vs. cases before TX (Chi-square test).

*a* indicates *p* < 0.05 in controls vs. cases before TX (unpaired t test or Mann-Whitney u test).

*b* indicates *p* < 0.05 in cases before vs. 1 month after TX (repeated measures ANOVA).

*c* indicates *p* < 0.05 in cases before vs. 6 months after TX (repeated measures ANOVA).

*d* indicates *p* < 0.05 in cases 1 vs. 6 months after TX (repeated measures ANOVA).

*ns:* non-significant.

HDL-associated PON1 paraoxonase and arylesterase activities were significantly lower in patients before transplantation compared to healthy controls; and these activities were slightly increased during the follow-up but did not reach the controls’ activities.

As it can be seen in Table 3, the percentage and the absolute amount of very low-density lipoprotein (VLDL) and small-dense LDL subfractions were significantly lower, while the percentage and the amount of intermediate density lipoprotein (IDL) subfraction were higher in healthy controls compared to patients before transplantation. One month after TX, the percentage and the amount of small-dense LDL subfraction decreased, while the percentage and the amount of large LDL subfraction increased after 6 months follow-up in patients (calculated by repeated measures ANOVA).

**Table 3.** LDL subfraction analysis by Lipoprint® in study individuals.

	Controls	Cases before TX	Cases after 1 month TX	Cases after 6 months TX	*p* values
VLDL %	17.4 ± 2.2	23.6 ± 3.7	23.1 ± 3.2	22.67 ± 3.8	*a, c*
IDL %	29.2 ± 4.9	26.3 ± 4.9	25.6 ± 4.6	24.6 ± 3.9	*a, c*
Large LDL %	21.5 ± 6	20.4 ± 4.8	22.3 ± 5.5	23.6 ± 4.7	*b, c*
Small-dense LDL %	0.6 (0–0.8)	1.2 (0–3.95)	0 (0–1.3)	1.3 (0–1.7)	*a, b*
VLDL mmol/l	0.88 ± 0.17	1.1 ± 0.36	1.32 ± 0.36	1.22 ± 0.38	*a, b, d*
IDL mmol/l	1.48 ± 0.38	1.25 ± 0.48	1.48 ± 0.46	1.32 ± 0.42	*b, d*
Large LDL mmol/l	1.09 ± 0.36	0.97 ± 0.4	0.63 ± 0.79	1.28 ± 0.45	*b, c, d*
Small-dense LDL mmol/l	0.02 (0–0.05)	0.05 (0–0.198)	0 (0–0.077)	0.059 (0–0.097)	*a, b*

IDL: intermediate density lipoprotein; LDL: low-density lipoprotein; TX: kidney transplantation; VLDL: very low-density lipoprotein. Data are presented as mean ± standard deviation or median (upper-lower quartiles).

*a* indicates *p* < 0.05 in controls vs. cases before TX (unpaired *t* test or Mann–Whitney *U* test).

*b* indicates *p* < 0.05 in cases before versus 1 month after TX (repeated measures ANOVA).

*c* indicates *p* < 0.05 in cases before versus 6 months after TX (repeated measures ANOVA).

*d* indicates *p* < 0.05 in cases 1 versus 6 months after TX (repeated measures ANOVA).

Similar to total HDL-C, the amount of intermediate and small HDL subfraction was significantly higher in controls compared to patients before TX. During the follow-up, these previously mentioned subfractions significantly increased in patients and reached the healthy control’s level (calculated by repeated measures ANOVA, see Table 4).

**Table 4.** High-density lipoprotein (HDL) subfraction analysis by Lipoprint ® in study individuals.

	Controls	Cases before TX	Cases after 1 month TX	Cases after 6 months TX	*p* values
Large HDL %	29.9 ± 9.2	30 ± 10.9	34.5 ± 13.9	28.7 ± 8.3	*d*
Intermediate HDL %	50.6 ± 4.7	48.8 ± 5	48.7 ± 5.7	50 ± 4.8	*ns*
Small HDL %	19.5 ± 5.6	21.2 ± 7.3	18.3 ± 7.7	21.2 ± 7.2	*D*
Large HDL mmol/l	0.49 ± 0.29	0.39 ± 0.23	0.77 ± 0.19	0.45 ± 0.27	*B*
Intermediate HDL mmol/l	0.75 ± 0.15	0.55 ± 0.15	0.55 ± 0.26	0.74 ± 0.42	*a, c, d*
Small HDL mmol/l	0.28 ± 0.06	0.22 ± 0.08	0.28 ± 0.12	0.29 ± 0.2	*a, b, c*

TX: kidney transplantation. Data are presented as mean ± standard deviation.

*a* indicates *p* < 0.05 in controls versus cases before TX (unpaired *t* test or Mann–Whitney *U* test).

*b* indicates *p* < 0.05 in cases before versus 1 month after TX (repeated measures ANOVA).

*c* indicates *p* < 0.05 in cases before versus 6 months after TX (repeated measures ANOVA).

*d* indicates *p* < 0.05 in cases 1 versus 6 months after TX (repeated measures ANOVA).

*ns*: non-significant.

There was a correlation between PEDF level and BMI in TX patients (*r* = 0.37; *p* = 0.004). Age did not show any correlations with the concentration of PEDF (controls: *p* = 0.64 and TX patients: *p* = 0.56).

**Figure 1.**
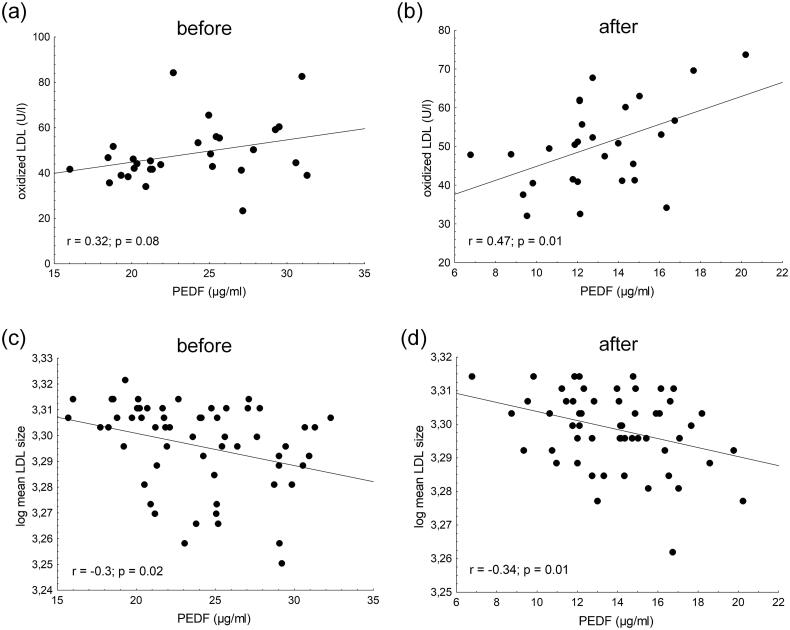
Pearson correlations between serum pigment epithelium derived-factor (PEDF) and (a) baseline oxidized LDL; (b) oxidized LDL concentration 6 months after kidney transplantation; (c) baseline mean LDL size and (d) mean LDL size 6 months after kidney transplantation.

**Figure 2.**
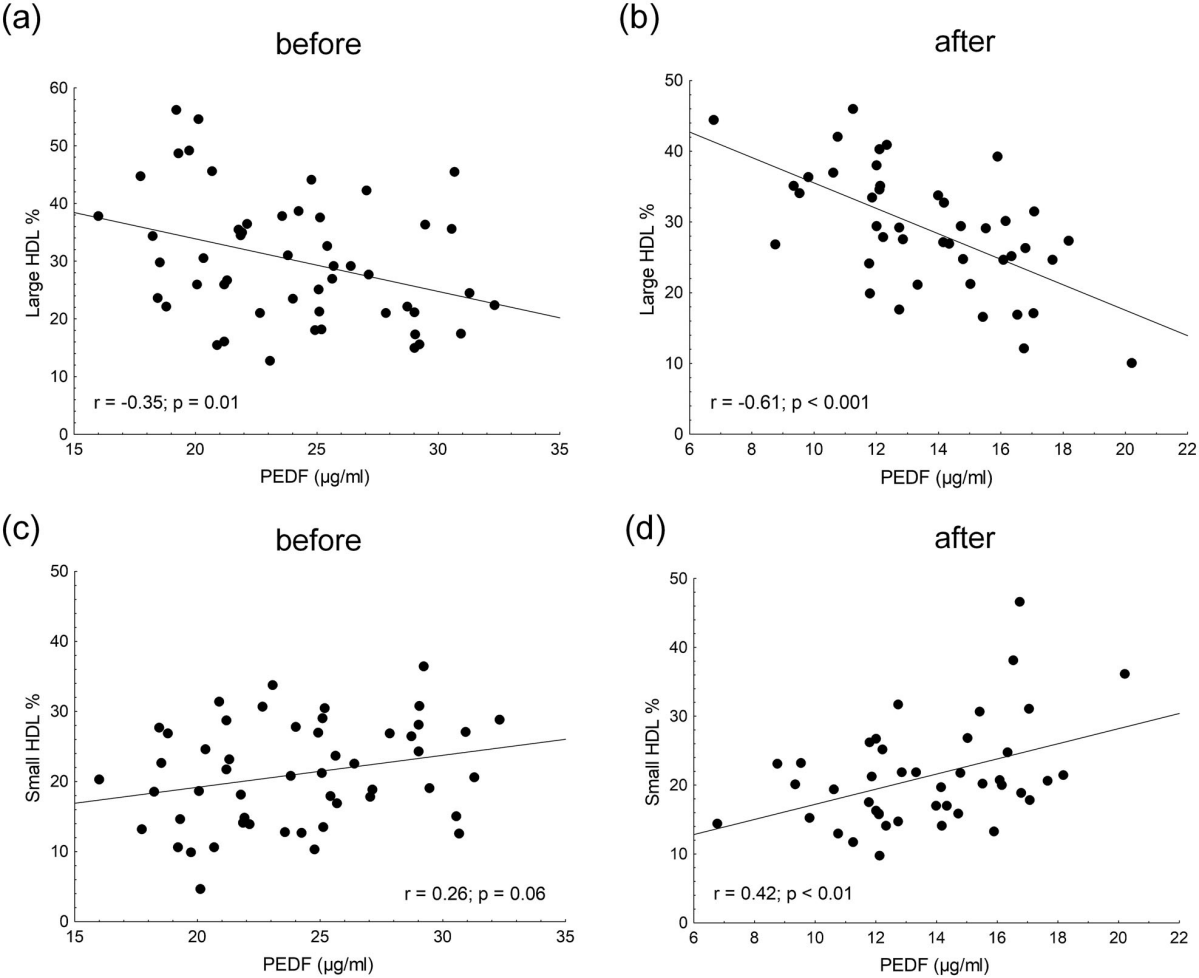
Pearson correlation between serum pigment epithelium derived-factor (PEDF) and (a) percentage of baseline large HDL; (b) percentage of large HDL 6 months after kidney transplantation; (c) percentage of baseline small HDL and (d) percentage of small HDL 6 months after kidney transplantation.

The pearson correlations between circulating concentrations of PEDF and various lipid parameters in transplanted patients before and after renal transplantation are summarized in Table 5. Before TX, only mean LDL size, triglyceride, percentage of VLDL and percentage of large and intermediate HDL subfractions correlated with the PEDF level. During the follow-up, other lipid parameters showed correlations with the concentration of PEDF. The oxLDL level positively correlated with PEDF after 6 months follow-up, while mean LDL size showed a significant negative correlation with PEDF before and 6 months after renal transplantation (Figure 1). Analyzing the associations between PEDF and HDL subfractions, negative correlations were found between the percentage of large HDL and PEDF level before and 6 months after transplantation; while a positive correlation was established between the percentage of small HDL subfraction and the PEDF level at the 6-months follow-up (Figure 2).

**Table 5.** Pearson correlations between pigment epithelium derived-factor (PEDF) and lipid parameters in transplanted patients before and after kidney transplantation (TX).

	Cases before TX	Cases after 1 month TX	Cases after 6 months TX
Cholesterol (mmol/l)	*r* = 0.02; *p* = 0.8^ns^	***r* = 0.3; *p* = 0.02**	***r* = 0.44; *p* < 0.001**
HDL-C (mmol/l)	*r*= −0.2; *p* = 0.1^ns^	*r*= −0.1, *p* = 0.4^ns^	*r*= −0.1; *p* = 0.4^ns^
LDL-C (mmol/l)	*r* = 0.07; *p* = 0.06^ns^	*r* = 0.17; *p* = 0.2^ns^	***r* = 0.39; *p* < 0.01**
Oxidized LDL (U/l)	*r* = 0.3; *p* = 0.09^ns^	***r* = 0.52; *p* < 0.01**	***r* = 0.47; *p* = 0.01**
Log mean LDL size	***r*=**−**0.3; *p* = 0.02**	***r*=**−**0.32; *p* = 0.01**	***r*=**−**0.34; *p* = 0.01**
Log triglyceride	***r* = 0.44; *p* < 0.001**	***r* = 0.52; *p* < 0.001**	***r* = 0.31; *p* = 0.02**
VLDL %	***r* = 0.31; *p* = 0.02**	*r* = 0.2; *p* = 0.08^ns^	***r* = 0.35; *p* < 0.01**
IDL %	*r*= −0.2, *p* = 0.2^ns^	*r*= −0.24; *p* = 0.06^ns^	*r* = 0.06; *p* = 0.9^ns^
Large LDL %	*r* = 0.06; *p* = 0.6^ns^	*r* = 0.22; *p* = 0.09^ns^	***r* = 0.26; *p* = 0.05**
Log small-dense LDL	*r* = 0.08; *p* = 0.6^ns^	*r* = 0.1; *p* = 0.6^ns^	***r* = 0.42; *p* = 0.02**
VLDL mmol/l	*r* = 0.2; *p* = 0.1^ns^	***r* = 0.36; *p* < 0.01**	***r* = 0.5; *p* < 0.001**
IDL mmol/l	*r*= −0.09; *p* = 0.5^ns^	*r* = 0.1; *p* = 0.4^ns^	***r* = 0.34; *p* = 0.01**
Large LDL mmol/l	*r* = 0.1; *p* = 0.5^ns^	*r* = 0.01; *p* = 0.9^ns^	***r* = 0.46 *p* < 0.001**
Log small-dense LDL	*r* = 0.1; *p* = 0.5^ns^	*r* = 0.15; *p* = 0.5^ns^	***r* = 0.55; *p* < 0.001**
Large HDL %	***r*=**−**0.35; *p* = 0.01**	***r*=**−**0.32; *p* = 0.02**	***r*=**−**0.61; *p* < 0.001**
Intermediate HDL %	***r* = 0.37; *p* < 0.01**	*r* = 0.1; *p* = 0.4^ns^	***r* = 0.42; *p* < 0.01**
Small HDL %	*r* = 0.26; *p* = 0.07^ns^	***r* = 0.51; *p* < 0.001**	***r* = 0.42; *p* < 0.001**
Large HDL mmol/l	***r*=**−**0.34; *p* = 0.02**	***r*=**−**0.4; *p* < 0.01**	***r*=**−**0.53; *p* < 0.001**
Intermediate HDL mmol/l	*r*= −0.05; *p* = 0.7^ns^	*r*= −0.19; *p* = 0.2^ns^	*r* = 0.05; *p* = 0.7^ns^
Small HDL mmol/l	*r* = 0.15; *p* = 0.3^ns^	***r* = 0.44; *p* < 0.001**	*r* = 0.2; *p* = 0.1^ns^

Bold values highlights the significant correlations.

*ns*: non-significant.

A multiple regression analysis was performed in backward manner to determine the variables best predicting the PEDF level in transplanted patients 6 months after renal transplantation. The model included BMI, oxLDL, mean LDL size, cholesterol, triglyceride, VLDL, IDL, large LDL, small-dense LDL, large HDL, and small HDL levels. According to this analysis best predicting variable of the PEDF level was the large HDL level (*β*= −0.58; *p* < 0.01).

## Discussion

The present paper is the first follow-up study reporting changes in serum PEDF levels of patients with chronic renal failure after kidney transplantation. Before transplantation, our patients had a significantly higher PEDF level compared to controls, similar to some previous reports [18,20]. We have found that 1 month after transplantation the PEDF level markedly decreased reaching the healthy controls’ level, and this low level was maintained during the 6 months follow-up period. Considering the anti-atherogenic effects of PEDF in chronic renal failure, this significant drop in the circulating PEDF levels may be unfavorable for our patients and may contribute to the high cardiovascular morbidity and mortality. Our results may also indicate that PEDF could constitute a therapeutic target and/or agent after kidney transplantation. On the other hand, the PEDF/creatinine ratio – which was calculated in order to standardize the PEDF levels based on the knowledge that PEDF might change with renal function – was 80% lower in cases before TX compared to controls. However, this ratio significantly increased after one month, but still remained only a half of the controls’ value. Nevertheless, we were not able to find any significant correlations between PEDF and creatinine levels before or after transplantation, which may indicate an indirect relationship between PEDF levels and renal function.

Abnormalities of lipoprotein metabolism are common and may contribute to the high incidence of cardiovascular disease that complicates chronic renal failure and persists even following successful renal transplantation. A previous study reported that the total cholesterol was increased in 44% of renal transplant recipients, while the triglyceride was elevated in 33% and the HDL cholesterol reduced in 16% of transplant recipients. The presence of small LDL particles is a known feature of uremic dyslipidemia that may represent an important risk factor for cardiovascular disease. This abnormality is not corrected by hemodialysis and persists also after kidney transplantation [27]. It has also been demonstrated that renal transplant recipients presented significantly lower levels of HDL3a and HDL3b and, in males, higher levels of HDL2b than controls [28]. Our results confirm these data. Small-dense LDL subfractions were significantly higher in TX patients compared to healthy controls before transplantation. One month after TX, the percentage and the amount of small-dense LDL subfraction decreased; while after the 6-months follow-up the percentage and the amount of large LDL subfraction increased. The mean LDL size showed a significant negative correlation with PEDF before and 6 months after renal transplantation. Furthermore, the amount of intermediate and small HDL subfraction was significantly higher in controls compared to patients before TX. During the follow-up, these previously mentioned subfractions significantly increased in patients, but in our study they reached the healthy controls’ level. Negative correlations were found between the percentage of large HDL and PEDF level before and 6 months after transplantation, while a positive correlation was established between the percentage of small HDL subfraction and PEDF level after the 6-months follow-up. To test whether the associations detected in the univariate analyses were independent of lipid parameters, we carried out a multiple regression analysis with PEDF level as the dependent variable. Based on the backward stepwise analysis, the PEDF level turned out to be best predicted by large HDL subfraction concentration. The significant negative correlation between serum HDL and PEDF levels has been reported previously in a population including healthy subjects and patients with impaired carbohydrate metabolism. *In vitro* data have shown that an increased dosage of HDL reduced the secretion of PEDF in adipocytes. However, the mechanisms of regulating PEDF expression and secretion by HDL is not clarified yet [29]. A previous study showed that the administration of D-4F, an apolipoprotein A1 mimetic peptide might markedly attenuate the oxLDL-provoked decrease in PEDF protein and messenger ribonucleic acid (mRNA) expression in human umbilical vein endothelial cells [30]. It has been reported that PEDF might be associated with the HDL particle in healthy subjects and in patients with an end stage renal disease both before and after renal transplantation. Moreover, PEDF was found to be accumulated in HDL of end-stage renal failure patients compared to healthy controls, while the enrichment of PEDF in the transplant group was markedly reduced as opposed to patients with an end stage renal failure, especially in transplant patients with a good graft function. They concluded that the restoration of renal function after renal transplantation does not correct impairment of uremic HDL properties [31]. Our results are in line with these previous data. These significant correlations between PEDF levels and lipoprotein subfractions may highlight the direct interplay between lipoprotein and metabolism and endogenous antiangiogenic mechanisms.

The adverse effects of oxidative stress on kidney transplantation have been demonstrated by experimental studies in animals, observational evidence from population-based studies, and several controlled clinical trials [32]. In addition to adversely affecting the allograft function and structure, oxidative stress plays a key role in the pathogenesis of systemic inflammation, hypertension, cardiovascular disease and neoplasm among other complication in transplant recipients [33]. Kidney transplant recipients are prone to reperfusion injury and demonstrate continual oxidative stress during the early phase of transplantation [34]. Based on findings obtained from living donor transplant recipients show that improvement of oxidative stress parameters begins immediately after kidney transplantation and continues up to the 28th post-transplant day. A complete remission is only achieved when the kidney function becomes normal [35]. The imbalance between oxidant and antioxidant factors after kidney transplantation are well documented. Decreased activities of HDL-associated antioxidant enzyme PON1 in renal transplant patients were reported previously [36,37]. Based on our results, transplanted patients had significantly higher serum oxLDL compared to the controls, while PON1 activities were significantly lower in the patient group compared to the controls. Furthermore, after transplantation, oxLDL levels were found significantly increased both 1 and 6 months after transplantation. Both PON1 paraoxonase and arylesterase activities were slightly increased during the post-transplant follow-up. We found a significant positive correlation between the levels of PEDF and oxLDL, while there were no correlations between the PEDF level and PON1 activities. To date, the detailed inhibitory effects of PEDF on oxidative stress is not fully elucidated. It has been demonstrated that oxLDL led to the downregulation of PEDF in human umbilical vein endothelial cells, which may have been triggered by the oxLDL-induced promotion of reactive oxygen species (ROS) [30]. Another study has shown that PEDF attenuates endothelial injury by blocking the Wnt/β-catenin pathway, subsequently ameliorating oxidative stress [38]. Our results also suggest that PEDF may inhibit the oxidative stress by decreasing oxidative agents rather than inducing antioxidant capacity in transplant patients. On the other hand, it was reported that oxLDL down-regulates the PEDF expression through an increased oxLDL-induced intracellular production of ROS [30], while we found a positive correlation between PEDF and oxLDL. We speculate that the enhanced oxidative stress in both HD treated and TX patients may cause tissue injury, inflammation and dysfunction, which induces the expression of PEDF, leading to an increased serum level of PEDF despite the negative intracellular effect of ROS on PEDF expression, limiting PEDF overproduction. This hypothesis should be corroborated by further studies.

Donor-specific antibodies lead to adverse outcomes by injuring the graft endothelium. In patients with antibody-mediated rejection, elevated levels of endothelial transcripts have been found in the allograft tissue [39]. The presence of circulating DSA and elevated endothelial transcripts in the allograft were associated with poorer long-term graft survival [40], even when evidence for complement activation was lacking [39]. There was a significantly lower PEDF level in the DSA positive group before transplantation highlighting the possible role of PEDF in the immunological processes.

Some limitations of our study must be mentioned. Although patients with more than 1.5–2.0 L overhydration were excluded from our study, overhydration may have an effect on the initial serum PEDF levels, as reported by Liu et al. [41]. Moreover, a larger number of TX patients and control participants may enhance the statistical power.

## Conclusion

We have demonstrated that the serum PEDF level significantly decreased after renal transplantation. The serum PEDF level was best predicted by a large HDL subfraction concentration indicating the role of HDL composition in PEDF expression. Based on these results, the altered molecular composition of HDL after transplantation may directly contribute to the enhanced atherogenesis leading to premature cardiovascular complications. Endothelial injury in the allograft macro- or microvascular beds, especially when antibody-mediated, reduces graft survival. DSA-mediated endothelial damage can occur through both complement-dependent and independent pathways. The pathophysiological role of changes in PEDF levels after transplantation needs to be further studied. These data suggest that PEDF may be a therapeutic target for alleviating ox-LDL–induced vascular endothelial cell damages after renal transplantation.

## Data Availability

All data generated or analyzed during this study are included in this published article. All data generated or analyzed during the current study are available from the corresponding author on reasonable request.
